# Acceptability and feasibility of CHANGE, a non-specialist worker delivered intervention to address alcohol use disorders and psychological distress among conflict-affected populations in Uganda: a qualitative study

**DOI:** 10.1016/j.jmh.2025.100361

**Published:** 2025-09-20

**Authors:** Abhijit Nadkarni, Catharina Van der Boor, Jacqueline N. Ndlovu, Dalili Taban, Wietse A. Tol, Bayard Roberts, Helen A. Weiss, Josephine Akellot, Soumya Singh, Melissa Neuman, Carl May, Eugene Kinyanda, Daniela C. Fuhr

**Affiliations:** a Department of Population Health, London School of Hygiene and Tropical Medicine, London, UK; b Addictions and Related Research Group, Sangath, Goa, India; c Department of Health Services Research and Policy, London School of Hygiene and Tropical Medicine, London, UK; d Department of Public Health, University of Coppenhagen, Denmark; e HealthRight International, Mawanda Road -Kamwokya, Kampala, Uganda; f Department of Infectious Disease Epidemiology and International Health, London School of Hygiene and Tropical Medicine, London, UK; g MRC/UVRI Uganda Research Unit, London School of Hygiene and Tropical Medicine, Entebbe, Uganda; h Department of Prevention and Evaluation, Leibniz Institute for Prevention Research and Epidemiology, Bremen, Germany; i University of Bremen, Health Sciences, Bremen, Germany

**Keywords:** Task-sharing, Alcohol use disorders, Psychological distress, Conflict-affected population, Uganda, Qualitative study

## Abstract

**Introduction:**

CHANGE is a psychological intervention designed using a systematic intervention development process for addressing the needs of men with co-existing psychological distress and alcohol use disorders (AUD) in conflict-affected settings. The aim of this study in Uganda was to understand experiences of those who delivered and received the intervention to inform contextually relevant adaptations before testing its cost-effectiveness in a randomised controlled trial.

**Methods:**

The study was implemented in the Rhino Camp refugee settlement in Northern Uganda. We conducted three sequential sets of 10 individual semi-structured in-depth interviews each with (a) adult (≥18 years) men with hazardous/harmful drinking and psychological distress who received the CHANGE intervention, and (b) non-specialist workers (NSWs) who delivered the CHANGE intervention. Thematic analysis was used to analyse the data.

**Results:**

The experiences of the men with hazardous/harmful drinking and NSWs were broadly consistent with each other. The participants found the knowledge and skill acquisition related to alcohol use, and the intervention materials such as handouts useful. Feasibility of the intervention was enhanced by its structured nature with in-built flexibility, and intervention content was perceived as easily comprehensible. On the other hand, the loss of potential earnings due to time spent in the sessions was a barrier to attendance. The intervention was perceived to result in reduced drinking and improvement in related activities such as sleep, appetite, and social relationships. Some of the perceived mechanisms for change included distraction and strengthening of supportive social networks. The day-to-day challenges of life in a refugee camp were a common barrier to changing drinking behaviour despite receiving the intervention.

**Conclusion:**

If proven to be effective, the acceptability and feasibility of CHANGE makes it a potentially scalable intervention in low resource settings with shortage of specialist healthcare professionals. The intervention may have the potential to be integrated with other programmes of care that can address additional adversities that the population may face in the setting.

## 1 Introduction

Approximately one in five people in conflict-affected settings experience mental health problems (Charlson et al., 2019), much higher than the mean global prevalence of one in 14 (Vos et al., 2017). Among internally displaced persons and refugees, the prevalence of hazardous/harmful alcohol use may range from 17 %−36 %, with a greater prevalence in males than in females (Horyniak et al., 2016). Mental disorders such as anxiety, depression, and post-traumatic stress disorder (PTSD) (Anker and Kushner, 2019; Hawn et al., 2020; McHugh and Weiss, 2019) are closely linked with alcohol use disorders (AUD). This relationship is consistently identified among the general population and conflict-affected populations (Hou et al., 2020).

Mental health and psychosocial support (MHPSS) guidelines for humanitarian settings, such as the Inter-Agency Standing Committee guidelines (Inter-Agency Standing (IASC) 2006), recommend action to address AUD. However, AUD interventions are typically not included in humanitarian responses (Greene et al., 2018), which may be because of a lack of evidence-based and scalable interventions for AUDs in conflict-affected settings (Greene et al., 2019). Furthermore, existing AUD interventions for general populations are generally not adapted to the sociocultural context of conflict-affected populations, do not address co-existing psychological distress, and are designed to be delivered by mental health specialists of whom there are very few in such settings. Additional barriers to implement such programmes for people with AUDs in such settings are stigma, resource limitations, provider knowledge and capacity, instability, disruptions to health systems, and attrition of healthcare workers, resulting in a high treatment gap (Greene et al., 2018).

One way of overcoming these barriers is deploying low-intensity psychological interventions delivered through task-sharing with non-specialist workers (NSWs). Task sharing involves the strategic redistribution of tasks, usually from highly trained health workers to those with shorter training or fewer qualifications, including laypeople (World Health Organization 2007). NSWs can be conceptualised based on their background and level of training as lying on a spectrum with the ‘natural helper’ (unpaid community members) at one end and the ‘para-professional’ (paid workers with minimal qualifications, trained and demonstrating acceptable levels of standardised competencies) at the other (Eng et al., 1997). There is growing evidence demonstrating that psychological interventions delivered by a low-cost, widely available human resource have moderate to strong effects in reducing the burden of AUDs and common mental disorders (such as depression) among the general population in low- and middle-income countries (Nadkarni et al., 2023; Singla et al., 2017). However, adoption of such evidence-based interventions should be coupled with adaptations informed by culturally sensitive research to account for contextual influences such as cross-cultural variability, and health system characteristics.

Problem Management Plus (PM+) is a potentially scalable psychological intervention designed by the WHO to reduce psychological distress among people exposed to adversity (World Health Organization 2016). It is a trans-diagnostic intervention that can be delivered by non-specialist providers over five sessions and targets symptoms of anxiety and depression, using strategies such as problem-solving, stress management, behavioural activation, and social support. PM+ has been demonstrated to be effective in reducing psychological distress among populations in different contexts (Schäfer et al., 2023). However, PM+ currently does not contain psychological strategies that directly address co-occurring AUD.

The work described in this paper is a part of the AlCohol use in HumanitariAN settings: a programme of work to address alcohol use disorders and associated adversities among conflict-affected populations in UGanda and UkrainE (CHANGE) (Fuhr et al., 2021). Through a systematic process we developed CHANGE, a potentially contextually relevant psychological intervention to be delivered by NSWs for addressing the needs of people with co-existing psychological distress and AUD in conflict-affected settings. The multi-step intervention development process involved (Nadkarni et al., 2025): 1) identifying treatment strategies via a meta-review and Delphi survey with international experts on AUDs; 2) conducting in-depth interviews (IDIs) with stakeholders (men with drinking problems, family members of men with drinking problems, community leaders, healthcare workers) in Uganda and Ukraine; and 3) holding consultative workshops with AUD experts to create a theoretical framework for the intervention. The Delphi survey reached consensus on key intervention components, such as problem-solving skills, handling drinking urges, and communication skills. From the IDIs, factors like psychosocial stressors, cultural norms, and coping strategies were identified as influencing alcohol use. The intervention, developed through workshops is described below and consists of three phases: assessment and feedback (Phase 1), providing skills for handling high-risk situations (Phase 2), and relapse prevention (Phase 3).

Because of the political situation in Ukraine, work in Uganda has progressed at a faster pace and hence this paper only reports work from Uganda. The focus of the intervention were males with AUD. While all genders might be exposed to stressors during conflict, the types of stress exposure are often strongly gendered. The responses to the stress exposure are also gendered, reflecting broader societal roles and coping mechanisms. As a result, male gender is strongly associated with AUD among conflict-affected populations globally (Lo et al., 2017).

The CHANGE intervention has been developed by integrating various AUD related intervention components into Problem Management Plus (PM+) (World Health Organization 2016). The core strategies of PM+ include ‘managing stress’, ‘managing problems’, behavioural activation, and strengthening social support (Dawson et al., 2015). In addition to these strategies delivered to address psychological distress, the AUD-specific strategies include detailed assessment, personalised feedback, psychoeducation, goal setting and change planning, cognitive behavioural skills (e.g., drink refusal, problem solving) and management of potential or actual relapses. The CHANGE intervention is delivered over six sessions which is consistent with the requirements of potentially scalable psychological interventions (Sijbrandij et al., 2020). These six sessions are implemented across three phases, with each phase having two intervention sessions. The focus of the first phase is to build rapport with the client, helping the client understand how adversity may affect their wellbeing and that their drinking may be causing problems. In this phase, the NSW explores the client’s motivation to change their drinking problems and encourages them to change by using motivational interviewing skills. In the second phase, the NSW’s role is to encourage and facilitate the client to commit to their plans to change their drinking problems and teach strategies that are useful in dealing with distress such as managing stress, managing emotions, and learning to manage drinking. In the third phase, the NSW helps the client prepare for situations that may lead to lapse or relapse and teaches strategies that help the client deal with distress and risky situations related to drinking after the intervention has ended. The duration of each session is around 60–90 min.

As part of our intervention development process, we conducted a study to test the acceptability, feasibility of delivery, and perceived effectiveness of the CHANGE intervention. The aim of the study was to understand NSW’s and participants’ experiences of the CHANGE intervention in Uganda, and this paper describes results of the study.

While the CHANGE intervention was developed and tested in Uganda, lessons from mental health programs in one conflict-affected setting may be applicable to other similar settings because of similarities in challenges across many conflict zones, such as high levels of trauma, displacement, ongoing adversity, stigma, limited resources, and the need for community-based support (Nisa et al., 2024; Garry and Checchi, 2020). Recent UN documents have highlighted the critical importance of a focus on alcohol use in humanitarian settings, where implementation of evidence-based strategies is largely lacking. Successful strategies, like task-sharing, culturally adapted interventions, and building local capacity, have been commonly applied and scaled in other regions facing similar conditions (Ndlovu et al., 2024). Additionally, our findings may be relevant to other conflict settings because of shared issues like security concerns, access barriers, and the importance of resilience-building (Troup et al., 2021).

## 2 Methods

### 2.1 Design

Individual semi-structured in-depth interviews nested in a treatment cohort.

### 2.2 Setting

The study took place in northern Uganda, namely in Rhino Camp refugee settlement that is largely inhabited by South Sudanese refugees and currently hosts an estimated 139,000 displaced people (Operational Data Portal 2022). Specifically, this study was conducted in the villages of Ofua 3 and Eden 1 in Rhino Camp.

### 2.3 Sample

Individuals living in the study settings were eligible to receive the CHANGE intervention if they were adult (≥18 years) male refugees with a score of 8–19 on the Alcohol Use Disorder Identification Test (AUDIT) (Saunders et al., 1993) indicating hazardous/harmful drinking, and scoring >6 on the Kessler Psychological Distress Scale (K10) indicating psychological distress (Kessler et al., 2002). Both these tools have been validated for use in Uganda (Kuteesa et al., 2019; Naisanga et al., 2022). Only men were screened as the prevalence of alcohol use and AUDs is higher for men than for women in northern Uganda (Roberts et al., 2011).

NSWs were adult members of the Ugandan host community who were recruited through advertisements in local newspapers, online platforms, and through word of mouth. NSW had to be members of the local community, should be at least 18 years old, completed high school, possess relevant experience in psychosocial or community work, be proficient in Juba Arabic and English, and show a desire to help others. Selection was also based on their performance during an interview and a role-play exercise After recruitment into the program, they received a nine-day training to deliver the CHANGE intervention, followed by supervised intervention delivery before the treatment cohort.

A subset of individuals who received the CHANGE intervention in a treatment cohort were selected for interviewing using an online random number generator after each phase of intervention delivery. In addition, all NSWs who delivered the intervention were invited to participate in the interviews. Interviews were conducted with five individuals who received CHANGE and five NSWs after completing each phase of the CHANGE intervention, resulting in three sets of 10 interviews each. Participating in an interview in one phase did not exclude the individual from random selection for another interview in the subsequent phases. The sampling strategy was designed to generate an understanding of acceptability and feasibility of delivering and receiving the intervention at different stages of the treatment journey. When deciding the sample size, our focus was on ‘information power’ which refers to the richness, relevance, and depth of the information gathered and how it contributes to the overall understanding of the phenomenon being studied (Malterud et al., 2016).

### 2.4 Procedures

Eligible participants were contacted via telephone by the local project coordinator and invited to participate in-person semi-structured interviews. The interviews of the recipients of CHANGE took place in a location of their choice near their homes and were conducted by trained research assistants. Interviews were conducted either in English or Juba Arabic, depending on the participants’ preference. The interviews of the NSWs were conducted in the HealthRight office in Arua. These interviews were conducted in English using a topic guide (Appendix 1) by a post-doctoral researcher (CB). All interviews were audio-recorded, transcribed, and translated into English where necessary. Interviews with both sets of participants lasted an average of approximately 40 min. All participants were compensated for their time with a bar of soap. All interviews were conducted between August and September 2022.

To reduce interviewer bias and encourage honest responses we rigorously trained the interviewer, used neutral and open-ended questions, stressed on establishing rapport to build trust with participants, assured confidentiality to encourage participants to provide authentic responses, and used a non-judgmental stance and active listening skills to ensure the data collected was rich and unbiased.

### 2.5 Analysis

Thematic analysis was used to analyse the data as described by Braun and Clarke (2006) (Braun and Clarke, 2006). As a first step, JN read through all interview transcripts to familiarise herself with the dataset and developed a preliminary coding framework inductively. Two co-authors (AN and DF) reviewed the preliminary coding framework and provided feedback. Preliminary themes were then abstracted from individual codes and further refined by JN. The final themes were mapped deductively onto corresponding themes: (i) Relevance of session content and acceptability of intervention, (ii) Feasibility of delivery, and (iii) Perceived effectiveness of session content. NVivo 14 was used to facilitate analysis.

The interviewer and coder had direct experience of the study setting, and neither of them were directly involved with the intervention delivery. The wider research team included senior-, mid-, and early-career researchers who contributed to the project in various capacities. These perspectives facilitated the interpretation of the data accounts as related to a priori theoretical and empirical foundations of complex intervention development and testing in the study settings.

### 2.6 Ethics

Ethical approval was provided by the London School of Hygiene and Tropical Medicine Ethics Committee (Ref: 26,560) and MildMay Uganda Research Ethics Committee (Ref: 0801–2022). All participants provided written consent for participation, and for the publication of anonymised results in scientific journals.

## 3 Results

Twenty men with drinking problems participated in the treatment cohort, of which 10 participated in the qualitative study. Fifteen NSWs delivered the CHANGE intervention in the treatment cohort, of which 11 participated in the qualitative study. The men with drinking problems who were interviewed for this study (called intervention participants from here on) were all refugees from South Sudan and their average age was 37.1 years (range 27 years to 52 years). All NSWs (7 female, 4 male) who were interviewed were from Uganda except one who was from South Sudan, and their average age was 31.9 years (range 26 to 37 years). For demographic characteristics of the NSW and intervention participants across the 3 phases see Table 1.

**Table 1:** Demographic Characteristics of the Non-Specialized Workers (NSWs) and Men Who Drink Across the Three Phases.

Phase	Sample	Age M (range)	Gender	Education	Nationality (ISO)	Displacement (Average years)
Phase 1						
	NSW (*N* = 5)	31.8 (30–33)	*F* = 2, *M* = 3	Diploma^a^=3 Bachelor’s degree=2	UGA=4 SSD=1	-
	Intervention participants (*N* = 5)	30 (25–36)	*M* = 5	Primary school= 2 Secondary school=2 Missing =1	SSD = 5	7
						
Phase 2						
	NSW (*N* = 5)	33.4 (28–41)	*F* = 4, *M* = 1	Diploma^a^=1 Bachelor’s degree=4	UGA=5	-
	Intervention participants (*N* = 5)	43.4 (29–52)	*M* = 5	Primary school =3 Secondary school=2	SSD=5	6
Phase 3						
	NSW (*N* = 5)	31 (28–34)	*F* = 4, *M* = 1	Diploma^a^=1 Bachelor’s degree=4	UGA=5	-
	Intervention participants (*N* = 5)	38 (25–52)	*M* = 5	Primary school=4 Secondary school=1	SSD=5	6

**Notes**:.

1. Sample includes individuals who participated in multiple phases.

2. A diploma is a mid-level qualification pursued after completing the Uganda Advanced Certificate of Education or its equivalent, prior to a bachelor’s degree.

3. Empty cells indicate missing data or fields not applicable to specific individuals.

### 3.1 Relevance of content and acceptability of intervention

#### 3.1.1 Knowledge and skill acquisition

The lack of knowledge on alcohol and its effects was consistently mentioned as a key contributor to drinking too much. Intervention participants highlighted how they often felt hopeless and overwhelmed and perceived the problems that they faced to be insurmountable. Coping with their situation through alcohol use was seen as the norm in the context. The adverse effects of drinking alcohol, including the effect on health, was not commonly understood. Hence acquiring knowledge about alcohol and its adverse impacts, and skills to reduce alcohol intake was perceived to be highly relevant for this context.

*“The most important thing for me in this session was that I realised drinking has more bad effects than good ones and also you may not leave drinking completely, but you can reduce the rate of drinking.”* (Intervention participant, age 32)

Intervention participants often mentioned that they were experiencing challenges in life in Uganda that they previously did not experience in South Sudan. These new, difficult experiences were found to be stressful and overwhelming, considering the limited resources at their disposal. They mentioned that the CHANGE intervention sessions helped them learn strategies to manage the problems that they face.

*“The part that talks about staying good with people and neighbours which actually makes me to stay well, the teachings made me to regain hope, made my mind to be okay, and it has made me to have that hope of having a strong heart even if things are not moving on well, it means it has strengthened my heart to face future issues that may arise”* (Intervention participant, age 27)

NSWs also documented some of the ways that intervention participants managed distress through setting attainable goals that directly address some of the challenges that they face.

*“I asked the client about his problems and then chose a particular problem and time, why it is happening, when, how and all those, so choose a problem like he had a pressing problem of his sons school fees that made him to drink alcohol and I asked him what he was going to do on this problem and told me that he was going to sell his crops in the garden like pumpkin in order to settle the problem which was the most challenging for him and it was understood, he also came with his action plan of selling his goat to handle the problem also.” (Female NSW, age 33)*

Intervention participants noted some unhelpful coping strategies that they engaged in previously, such as drinking alcohol to help them not ruminate about their problems. Additionally, they highlighted how the intervention helped them understand how coping through drinking alcohol negatively affected their productivity. Drinking had consequences on their ability to work, which in turn contributed to their stress as they were not able to earn money to support their families.

*“I took the advantage of leaving alcohol in order not to overthink because I was told that most of the thoughts come over because of too much drinking thus making one not to work.”* (Intervention participant, age 27)

#### 3.1.2 Intervention materials

As part of the intervention, the intervention participants were given handouts to take home with them. Overall, these handouts had content that was covered during the sessions. This was greatly appreciated, as the materials helped to reinforce what they had learnt during the sessions. The NSWs also used the handouts during sessions. They expressed how the pictorial content of the handouts was particularly helpful for clients with limited literacy skills and stressed that adaptations should be made to further increase inclusivity for clients with limited literacy skills.

*“I would like to request that more pictures should be added since we handle different clients both educated and uneducated so the pictures can clearly explain things” (Male NSW, age 30)*

#### 3.1.3 Unhelpful intervention content

Some of the elements of the intervention were found to be inappropriate. One such example, was the content related to setting of drinking goals and commitment to change. Both NSWs and intervention participants found this to be inappropriate and believed that it could lead to a socially desirable response rather than communicating true intent.

*“Where they asked me when are you going to leave or stop alcohol drinking. This question is not good, it puts you in difficult position, it can make one lie (…) At least give some time to monitor this person, let this question come after two weeks or a month when you have assessed whether the person has changed or not in terms of alcohol drinking.”* (Intervention participant, age 37)

*“That’s what I think for a client who is honest because there are those who can make up their mind and tell you that I am ready to reduce or stop drinking alcohol then you still want to bring out a ruler that says I want to be very sure at the scale of this to that, tell me where your readiness falls. This to some clients it feels like you don’t believe in them.”* (female NSW, age 33)

Additionally, NSWs expressed discomfort when asking clients for feedback on how to enhance the CHANGE program. This was because intervention participants consistently requested material or livelihoods assistance. Similarly, intervention participants voiced frustration as they did not feel adequately compensated monetarily for the time they invested in attending the program. Consequently, interviewees emphasized the importance of incorporating further skill development content into the program that could eventually lead to livelihood support.

*“We have a question that says, “what will we be improving on[the intervention]” as an intervention facilitator we are always asking this question but what always comes to the client’s mind are tangible supports that organizations can give them for example livelihood activities, here they feel the intervention must go hand in hand with livelihood activities.”* (Male NSW, age 30)

#### 3.1.4 Gender of delivery agents

Most NSWs and intervention participants did not see gender dynamics affecting intervention delivery. The priority for most of the men was to receive the sessions, and for them the gender of the NSW did not matter. However, some of them felt that having a male NSW helped them to open up more easily, particularly when coming up with strategies for managing conflict with their wives. The men felt that male NSWs were able to help them more because they expected them to have experienced similar challenges and hence understood them better.

*“Setting plans between men was very easy. This was because both of us were from the same gender and we easily understood each other because the intervention facilitator may also at some point experience the same problems that I experience for example the challenges of quarrelling with women at home. This made it easy for us to come up with solutions and ways forward. So, I think him being a fellow man like me was very important.” (Intervention participant, age 32)*

### 3.2 Feasibility

#### 3.2.1 Structure of the intervention

Overall, NSWs found the intervention easy to deliver, and intervention participants easily understood most of the concepts in the sessions. NSWs found delivering sessions easy, as they were continually guided by the manual. In situations where the strategies in the manual were deemed to be inappropriate to be delivered at a particular stage by the NSWs, they practiced flexibility to meet contextual requirements.

*“To some clients there is need to bring stress management which is in session three, but I brought it to session two because some of the clients appeared to be stressed during the interventions.”* (Male NSW, age 32)

At times, this created challenges with the time it took to deliver a session, however, the NSWs prioritised the needs of the participant.

*“Sometimes the sessions may go beyond the planned time just because (…) he gave me a specific feeling that I need to handle him with care like giving him a lot of time for psycho- education.”* (Male NSW, age 37)

Additionally, NSWs also adapted some aspects of the manual to enhance comprehensibility. They replaced technical terms from the manual with simpler terms, and used culturally appropriate examples that participants could relate to.

*“These are words that are hardly understood by the participants especially the term adversity is hard, so it’s easy when you explain it in Juba Arabic, and you break it down into simple terms as problems or challenges so that it is easier for client to focus in the session”. (Male NSW, age 32)*

### 3.2.2 Comprehensibility of intervention content and ease of session delivery

The level of education of the intervention participants influenced their understanding of some of the intervention content. For example, the calendar activity where they needed to plan out activities to meet their goals was better understood by those who had gone to school and had been exposed to schedules and planning. Additionally, NSWs who were native speakers of the local language found it easier to explain intervention strategies.

*“I really understood all that she* (NSW) *told me because the language she used to talk to me was very easy and I was able to understand her very well.”* (Intervention participant, age 31)

NSWs also noted that activities such as breathing exercises were sometimes not taken seriously by the men, especially those who were not experiencing acute distress. The exercise was initially perceived to be funny by some. However, after an explanation of how it worked and after participants actively practised it at home, the exercise came to be more appreciated.

*“The client was able to demonstrate it well to the extent of even doing it at his free time, he kept on practicing what he had learnt because on each and every session, I normally do review of what went well in the previous session*” (Male NSW, age 32)

The NSWs also noted that they themselves offered new examples to clients, and clients offered their own examples to make the content more accessible.

*“For instance the urge surfing, I gave him an example of the whirl-wind that moves during the dry season when it comes and finds you standing somewhere and you stood very strong, this wind will eventually not blow you off and moves away and in explaining this to the client, he was able to also come up with an example saying that this is in-line with when you want to eat a particular kind of thing and that thing is hard to get or found, what you need to do is to let the urge for that particular thing go.“* (Female NSW, age 35)

#### 3.2.3 Barriers to attendance

The men pointed out that by attending sessions, they were using time that they would have used to generate income for themselves and their families. They felt that the time spent on the intervention should be compensated for economically, to motivate them to keep attending the sessions. Although they learnt strategies to manage distress and to reduce alcohol consumption, the men also expressed needing material support that was tangible.

*“He told me, he appreciates it* (CHANGE intervention) *but I could see that he was no longer ready to sit because he said sitting for 1 hour and 30* min *without giving them anything and yet they could be doing other things to look for ways of supporting their families within that time.”* (Female NSW, age 29)

### 3.3 Perceived effectiveness of the intervention

#### 3.3.1 Change in drinking behaviour

The NSWs reported that some of their clients had reduced their alcohol consumption from the second session onwards. Intervention participants also reported reducing their drinking progressively after each session. One participant mentioned that he had not consumed any alcohol for two weeks, which he previously was unable to do. There was a general understanding across all participants that stopping alcohol consumption completely was a gradual process. While most set a goal to work towards stopping their drinking completely, some chose to reduce their consumption, with an understanding that at the reduced level of drinking it would not affect their ability to provide for their families.

*“In a week, I only drink once and no one realises that I drink, I provide for my family, and they have food in the house.”* (Intervention participant, age 32)

Some of the factors that were expected to influence the sustained effect of the intervention were use of other substances, and lack of livelihood and skill development in the intervention. Intervention participants felt that although they had been helped during the sessions, the likelihood of relapsing was very high because they would go back to being idle (risk factor for drinking) without any skills or livelihood activities. They suggested that the intervention could be integrated with livelihood activities or skills training for example, to help with generating income while also receiving psychosocial support. They believed that such an intervention could better improve their lives and keep them busy.

*“You will not help us in terms of other ways like giving us trainings and other skills that can help us change our wellbeing. I think will take us behind to our old level because I think after finishing the sessions, if still we don’t have anything to do, we will still go back to our original level* (of drinking)*.”* (Intervention participant, age 31)

#### 3.3.2 Changes related to drinking behaviours

While there was a consensus amongst the men that the intervention had changed their lives, in many cases, they were not able to provide specific details of this change. The few that did provide examples reported that reduction in alcohol consumption improved their sleep and appetite, ability to socialize better, and enhanced their self-care.

Another change that the men reported was the ability to save their earnings whenever they got job opportunities. Previously, they were using their earnings to purchase alcohol, but they were now saving money to take care of their families.

*“Because it’s not like before whereby when you get 500 shillings* (∼4000 shillings=1USD)*, you take it and go to the roadside and you come back without the money because you end up drinking so being able to save those 500 shillings, and I take it back home to be able to buy some sugar is what is important to me now.”* (Intervention participant, age 35)

#### 3.3.3 Mechanisms of change

While previously the men would spend their days at the trading centres with friends drinking alcohol, they now reported applying the strategies learnt during the sessions and found ways of keeping themselves busy during the day. They would only go to the trading centres in the evenings. Other men in this study kept track of their reduction in alcohol consumption by counting the number of bottles they used to drink and comparing to how many bottles they now drink.

As part of the intervention, participants were strongly encouraged to keep busy and active to reduce cravings and time spent overthinking. When they restarted some of the activities that they used to do in South Sudan such as playing football, they experienced positive changes in their mood.

*“When I was taken through the session, I felt I was killing myself by staying in the room not doing anything in order to handle my situation, but I have realised that it is good to work than staying idle… it in fact has raised my status and I can stay like a different person not like before.”* (Intervention participant, age 43)

For other men who were already active, they expressed that this part of the intervention (keeping oneself occupied) was redundant and unhelpful for them. There were also expectations that NSWs would help participants to keep busy and help them change their drinking behaviour, highlighting a misunderstanding and inaccurate expectation of the CHANGE intervention.

*“He said he would only get changes in his drinking if he got busy, for him getting busy means he would get a job; and expected that I would get him that job.”* (Female NSW, age 29)

Engaging in group activities also built participants’ social capital, and they expanded their support networks. As part of the intervention, the men mapped out their social support, where they can go to when in need. The exercise helped them to think of the trusted people and organisations around them who they can rely on for support. This guided exercise was particularly helpful for participants who initially thought that they had no support and could not identify who could form a social support system. It was also helpful for participants to identify who was a positive and who was a negative source of support in their lives, and to take the necessary steps to strengthen the positive social support that they had identified.

#### 3.3.4 Barriers to changing drinking behaviour

The day-to-day challenges experienced by the intervention participants were a common barrier to changing their drinking behaviour. These included not having enough land for farming, inadequate food for their families, inability to pay their children’s school fees, adjusting to new settings, loss of loved ones, loss of livelihoods, and inability to provide for their families. In many cases, these challenges were the primary causes of drinking, as drinking alcohol was a coping mechanism to forget their problems for some time.

*“Feelings on how my life was before the occurrence of the war in South Sudan and the properties that I had before the war sometimes makes me overthink and managing the stress sometimes is very hard.”* (Intervention participant, age 33)

Another key challenge faced was influence from friends, who often offered to buy the alcohol even when the men could not afford it themselves. The men were encouraged to avoid these friends when identifying negative and positive social support networks. Finally, psychological distress and the associated tiredness itself acted as a barrier to attending intervention sessions as the sessions were considered to be too long.

*“It affected our real time for the session because my participant was tired, and he also had a psychological torture though after coming back he looked for me to conduct the session, but he seemed tired, and he told me to reduce the time for the session.”* (Female NSW, age 26)

## 4 Discussion

Our study examines the acceptability, feasibility, and perceived effectiveness of CHANGE, a contextually relevant intervention for co-existing AUD and psychological distress delivered through task sharing in conflict affected settings. Both the knowledge and skills acquired, and intervention materials shared through CHANGE were found to be acceptable and relevant both by the intervention participants and the NSWs in the context of Northern Uganda. While there was no consensus on the utility of gender-matching, some of the content was found to be unhelpful or irrelevant to the context, resulting in changes being made to the intervention manual. The way the intervention and its content were structured was found to be comprehensible by intervention participants and easy to deliver by the NSWs. Both reduction in drinking and resulting improvement in other domains of their lives were described by the participants. Strategies from the intervention that were reported to be helpful included distraction and activation of social networks. Finally, financial stressors, peer pressure, and psychological distress continued to be barriers to changing drinking behaviour – and intervention participants recommended the integration of the intervention with livelihoods components. Overall, our findings in a South Sudanese refugee settlement in Uganda indicate that it is feasible to deliver such an intervention, that content is acceptable to most recipients, and is perceived to lead to positive change on alcohol use and associated behaviours.

Our findings are consistent with other literature that has synthesised the evidence on mental health programs implemented in conflict settings. There is growing – yet limited - evidence for the effectiveness of mental health programs in conflict settings (Greene et al., 2018; Greene et al., 2019). However, actual uptake is slow because of several barriers, both at the individual and systemic level. These include shortage of individuals with adequate mental health training, attrition of lay workers taught to provide evidence-based interventions, contextual instability that limits ideal implementation, lack of resources needed to expand primary care facilities to accommodate more and/or different patients, constraints on travel to receive services, lack of knowledge and/or stigma about mental health services, lack of trust in the quality of health services and treatment fidelity, low priority accorded to mental health issues by most governments, and financial constraints (Murray et al., 2014).

Our findings are also consistent with other literature that has reported on adaptations of mental health programs. There is growing evidence that psychosocial interventions that were adapted to the context and populations are more effective than non- adapted ones (Hall et al., 2016). Indeed, it has been argued that the adaptation process should consider not only revision of content but also amend delivery mechanisms taking account of the local health system and understanding of distress (Sangraula et al., 2021). Obtaining information on local idioms of distress and testing the acceptability or feasibility of an intervention is only possible with a qualitative design which we also have followed in our study. While every context and setting is different, there are parallels with other mental health care programmes that adapted their treatment before evaluating its effects (Perera et al., 2020). For example, for a group PM+ study, mechanisms of action were aligned with local understandings of distress to make it more meaningful for participants (Sangraula et al., 2021) while other psychological interventions implemented in low and middle-income countries focused on amending the language, using local metaphors and considered reducing the complexity of treatment for non-literate participants (Chowdhary et al., 2014).

In line with the findings, several changes were made to the CHANGE manual. For the structure and delivery, the stress management technique was revised such that it could be introduced flexibly to the patients in any CHANGE session. This addressed NSW’s feedback on difficulties delivering stress management techniques in the third session, as clients often present with significant distress early on. In terms of the language, as participants and NSWs described difficulty understanding the concept of an "urge," the revised manual included a clear definition and a locally relevant example. Additionally, the term ‘weekly calendar’ was also replaced with the term ‘plan of activities’ as the latter was more comprehensible, particularly to those with lower levels of literacy. Further, to address clients’ reporting feeling ‘tired’ during the sessions due the session length and content, the revised manual included an appendix on fun warm-up exercises for such situations and reiterated the flexibility in delivering CHANGE. Finally, to improve management of expectations, it was further elaborated that CHANGE does not provide economic assistance to clients, though clients may have improved economic opportunities because of improvements in mental health and reduced drinking.

Beyond accepting that AUDs are a significant problem in some conflict-affected settings, humanitarian guidelines have limited guidance on the means to address the problem. One of the likely reasons for the limited response to AUD by most humanitarian actors is the lack of a reliable evidence base on contextually relevant and cost-effective interventions (Roberts and Ezard, 2015). Hence an intervention such as CHANGE is critical, as it addresses a much-neglected problem in conflict settings and is sensitive and responsive to the contextual challenges.

While our findings about the acceptability, feasibility and perceived effectiveness are encouraging, there is one key lesson for the development of psychological interventions for similar settings. While the intervention participants found the knowledge and skills acquired through the intervention helpful, a strongly endorsed theme was around the inability of the intervention to address the stressors related to livelihood and material resources that lead to the development and continuation of the drinking. Additionally, the same financial stressors and constraints acted as practical barriers to engaging with treatment. Some strategies to address the financial barrier include providing tele-therapy (e.g. via phone call), flexible scheduling outside of working hours, collaboration with social services and community organisations which can provide material support, implementing holistic care models that address both mental health and social needs, and policy advocacy.

A compelling explanation for the vulnerability to AUDs in conflict settings is cumulative socioeconomic disadvantage, which can multiply, sometimes exponentially, over the course of time, leading to adverse outcomes (Ezard et al., 2011). While there are many existing alcohol interventions that have been shown to be effective, not many (including CHANGE) have been implemented with the specific goal of addressing the social determinants that lead to AUDs. Further research on integrated, multi-sectoral interventions that can address both psychological distress and social determinants simultaneously is a key consensus-based research priority for mental health and psychosocial support in humanitarian settings (Tol et al., 2023). The current study provides a critical step towards such a research agenda, by improving understanding concerning feasible and acceptable psychological intervention components. While it is beyond the scope of our program, future iterations of our intervention should consider addressing structural issues like unemployment and economic hardship by integrating livelihood support, offering vocational training, microfinance, and job readiness skills. These initiatives can empower individuals economically, reduce financial stress, improve mental well-being, and enhance economic resilience, ensuring lasting impact.

This qualitative evidence helps to advance our understanding of the acceptability and feasibility of CHANGE, and this approach is essential for deployment of services that are responsive to the context. The strength of our qualitative research design is that it offers in-depth insights into the experiences, behaviours, and social lives of our participants by focusing on context and individual perspectives through rich, descriptive data. This is a particular strength as it allowed us to explore a complex, under-researched and stigmatised condition while capturing diverse, and nuanced experiences.

We acknowledge study limitations related to the nature of qualitative research and these include the subjective nature of the analysis influenced by the positionality of the researchers. Our sample size is consistent with qualitative studies where the focus is on the in-depth understanding of a phenomenon. Furthermore, we sampled more than half of the sampling universe, which is a substantial proportion for a process evaluation. The other key point to remember is that we did not select participants based on their outcome status (improved or not), and hence expect their responses to be influenced by their clinical status. Nonetheless, 63 % of men with drinking problems that were involved in the treatment cohort were interviewed. Finally, qualitative studies such as ours have limitations that can affect the interpretation of findings. Reliance on self-reported data introduces potential biases such as social desirability or memory recall issues, which may distort the accuracy of responses. Additionally, the limited generalisability of qualitative research, often due to small, homogenous samples or context-specific findings, restricts the applicability of results to broader populations. Future research on the CHANGE intervention could address some of these limitations by incorporating mixed-methods approaches, using larger and more diverse samples, and ensuring triangulation with other data sources to enhance the validity and reliability of findings.

Through its delivery by NSWs, CHANGE is potentially scalable in low resource settings with shortage of specialist healthcare professionals. Considering the feasibility and acceptability of CHANGE and the encouraging change in some outcomes in the positive direction, research on the cost-effectiveness of CHANGE is warranted. If cost-effective, such a contextually appropriate intervention could be a substantial achievement as it is the first comprehensive intervention for co-existing AUD and psychological distress. In addition to testing cost-effectiveness to ensure resources are used efficiently, future research on CHANGE should adapt it for diverse populations by considering cultural, gender, and socio-economic factors, and test its integration into multi-sectoral programs like education, livelihood, and social protection. In conclusion, our findings warrant further research on CHANGE as it has the potential to bridge the significant treatment gap for AUDs in conflict-affected settings.

Eventually the long-term viability and sustainability of such programs is dependent on several factors. These include strategies such as integrating mental health services into existing systems like primary health care, education, and livelihood programs, training local mental health professionals, promoting community-based interventions, leveraging cross-sector collaborations to address both psychological and material needs, securing diversified funding sources, and policy integration (Ndlovu et al., 2024). In summary, flexibility and adaptability to changing circumstances, along with ongoing advocacy for mental health in humanitarian responses, will help ensure these programs are resilient and sustainable over time. These avenues are currently being investigated in a follow-up study on the potential for scaling up CHANGE.

In conclusion, next steps for CHANGE include testing its cost-effectiveness in the setting for which it was developed (currently underway in Uganda), examining its effectiveness in a different setting (currently underway in Ukraine), and integration of the intervention into broader multisectoral programs responding to social determinants and impacts of AUDs in conflict settings. In addition, future research should aim to answer questions on how interventions of different resource-intensity levels may be combined in multi-layered packages (e.g., through stepped care models).

## Glossary

AUD: Alcohol use disorders

AUDIT: Alcohol Use Disorder Identification Test

CHANGE: AlCohol use in HumanitariAN settings: a programme of work to address alcohol use disorders and associated adversities among conflict-affected populations in UGanda and UkrainE

K10: Kessler Psychological Distress Scale

MHPSS: Mental health and psychosocial support

NSW: Non-specialist worker

PM+: Problem Management Plus

PTSD: Post-traumatic stress disorder

## Acknowledgements

None

## Funding source

This work was supported by the National Institute for Health Research (NIHR) (using the UK’s Official Development Assistance (ODA) Funding) and Wellcome (grant reference number 219468/Z/19/Z) under the NIHR-Wellcome Partnership for Global Health Research. The funder did not have any role in study design, in the collection, analysis and interpretation of data, in the writing of the report, and in the decision to submit this manuscript for publication.

## Ethics

This study has received ethical approval from the London School of Hygiene and Tropical Medicine Ethics Committee (Ref: 22729) and MildMay Uganda Research Ethics Committee (Ref: 0212–2020). All participants have provided written consent to participate, and for the publication of anonymised results in scientific journals.

## CRediT authorship contribution statement

**Abhijit Nadkarni:** Writing – original draft, Methodology, Conceptualization, Writing – review & editing, Supervision, Funding acquisition. **Catharina Van der Boor:** Investigation, Writing – review & editing. **Jacqueline N. Ndlovu:** Writing – review & editing, Formal analysis, Writing – original draft. **Dalili Taban:** Project administration, Writing – review & editing, Investigation. **Wietse A. Tol:** Writing – review & editing, Methodology, Conceptualization, Supervision, Funding acquisition. **Bayard Roberts:** Writing – review & editing, Funding acquisition, Methodology, Conceptualization. **Helen A. Weiss:** Writing – review & editing, Funding acquisition, Methodology, Conceptualization. **Josephine Akellot:** Writing – review & editing, Investigation. **Soumya Singh:** Writing – review & editing, Investigation. **Melissa Neuman:** Writing – review & editing, Data curation. **Carl May:** Methodology, Writing – review & editing, Conceptualization. **Eugene Kinyanda:** Methodology, Writing – review & editing. **Daniela C. Fuhr:** Writing – review & editing, Methodology, Conceptualization, Supervision, Funding acquisition.

## Declaration of competing interest

None.

## Appendix Supplementary materials

Image, application 1
