# Supplementary material for: Acceptability and feasibility of CHANGE, a non-specialist worker delivered intervention to address alcohol use disorders and psychological distress among conflict-affected populations in Uganda: a qualitative study
Source: J Migr Health. 2025 Sep 20;12:100361. doi: 10.1016/j.jmh.2025.100361 (PMC12510219; doi:10.1016/j.jmh.2025.100361)
Supplement: Supplementary file 1 [file mmc1.docx]

# **Appendix 1: Interview Guides**

# TREATMENT COHORT – PHASE I (session 1 and session 2)

**PM+A Post-Session Debrief: FACILITATOR**

[**INSTRUCTIONS**]

These questions are asked by the Researcher to the PM+A facilitator, immediately after each phase. We ask these questions to make sure we understand how people experience facilitating the sessions. The questions are about (1) the *relevance* of the session content; (2) *cultural acceptability* of the session content; (3) *comprehensibility* of the session content; and (4) the *perceived effectiveness* of the session content. The questions should be answered for all the session parts.

Please make sure you have completed the consent form with the facilitator.

**Phase I**

**I would like to ask you about each of the two sessions you have taken part in.** Before we start, I would like to emphasize two things. First, these questions are to make sure that the intervention is easily understood. The questions are not meant as a test (for example, like in school). We would really like to hear your own opinion.

**Session 1**

**In session one you talked to participants about PM+A, and particularly about adversity.**

[1.1] Were there parts that you think were helpful for the participants, or not?

Which parts were helpful? And why?

Which parts were not helpful? And why?

[1.2]. Were there parts that you felt are not relevant for people living here/people in similar circumstances as the participants? e.g., because they don’t address the specific problems they experience.

If so, which parts?

Why are these parts not relevant?

[1.3] Did you do anything differently from how it was specified in the manual? If yes, what did you do differently and what was the reason?

|  |
| --- |

[1.4] Was there any strategy or activity that you struggled to explain to the participants?

| If yes, what was it about these strategies or activities that made it difficult to explain?  What changes would you make to make these strategies or activities easier? |
| --- |

[1.5] What strategy or activity did you find easy to explain to participants?

[1.6] Would there be anything that you would change in this session overall? If so, what changes would you make?

If yes, why would you make these changes?

[1.7] Were you able to deliver the intervention session in the planned time? If not, why not?

**Session 2**

**In session two you talked to participants about their AUDIT scores, psychoeducation on drinking, identifying Pros and Cons, and Goal setting.**

Fi darasa nimira itnin, ita wonsu ma musharaka fogo natija ta AUDIT, Talim fogo asurub merisha, ayinu kwes wu batal tou wu kutu araada.

[2.1] Were there parts that you think were helpful for the participants, or not?

Which parts were helpful? And why?

Which parts were not helpful? And why?

[2.2]. Were there parts that you felt are not relevant for people living here/people in similar circumstances as the participants? e.g., because they don’t address the specific problems they experience.

If so, which parts?

Why are these parts not relevant?

[2.3] Did you do anything differently from how it was specified in the manual? If yes, what did you do differently and what was the reason?

|  |
| --- |

[2.4] What was your experience of delivering and explaining the strategies in this session?

|  |
| --- |

[2.5] What was your experience of using the CHANGE plan worksheet?

[2.6] Do you think the worksheet accurately reflects the lessons learned in this session?

[**READ OUT LOUD**]

[2.7] Were you able to deliver the intervention session in the planned time?

|  |
| --- |

[2.8] Would there be anything that you would change in this session overall? If so, what changes would you make?

If yes, why would you make these changes?

Thank you.

**Researcher ID:**

**Participant ID:**

**Date:**

**Start time:**

**End time:**

**TREATMENT COHORT – PHASE I (session 1 and session 2)**

**PM+A Post-Session Debrief: Participant**

[**INSTRUCTIONS**]

These questions are asked by the researcher to the PM+A participant, immediately after each phase. We ask these questions to make sure we understand how people experience the sessions. The questions are about (1) the *relevance* of the session content; (2) *cultural acceptability* of the session content; (3) *comprehensibility* of the session content; and (4) the *perceived effectiveness* of the session content. The questions should be answered for all the session parts.

Phase I

I would like to ask you about the two sessions you have taken part in. Before we start, I would like to emphasize two things. First, these questions are to make sure that the intervention is easily understood. The questions are not meant as a test (for example, like in school). We would really like to hear your own opinion. Second, it is fine if you do not understand everything perfectly now. Our experience has been that sometimes parts of the session only makes sense after some time.

First, I’d like to ask you about your overall impressions of the first two sessions of PM+A

[1.1] Can you describe your experience of participating in the PM+A program so far?

[1.2] Have there been any parts that have particularly stood out to you? i.e. that have been of interest to you?

[1.3] Were there parts that you felt are not relevant for people living here/people in similar circumstances as you?

If so, which parts?

Why are these parts not relevant?

[1.4] Were there parts that you felt were inappropriate?

Session 1

In session one we talked about PM+A, and particularly about adversity.

[2.1] Did anything stand out to you in terms of the conversations you had with your facilitator about adversity?

If so, what stood out to you?

Why did this stand out to you?

[2.2] Was there anything that you found difficult to understand?

[2.3] Were there parts of this session that you think were particularly helpful for you, or not?

Which parts were helpful? And why?

Which parts were not helpful? And why?

Thank you. Now, let’s talk about the specific parts of today’s session (session 2). In today’s session you focused on the pros and cons of drinking, goal setting and developing a change plan.

Session 2

[3.1] Did anything stand out to you in terms of the conversations you had with your facilitator concerning alcohol?

[3.2] Was there anything that you found difficult to understand today?

[3.3] Were there specific parts of this session that you think were helpful for you, or not?

Which parts were helpful? And why?

Which parts were not helpful? And why?

Thank you very much for your answers today.

# TREATMENT COHORT – PHASE II (session 3 and session 4)

**PM+A Post-Session Debrief: FACILITATOR**

[**INSTRUCTIONS**]

These questions are asked by the researcher to the PM+A facilitator, immediately after each phase. We ask these questions to make sure we understand how people experience facilitating the sessions. The questions are about (1) the *relevance* of the session content; (2) *cultural acceptability* of the session content; (3) *comprehensibility* of the session content; and (4) the *perceived effectiveness* of the session content. The questions should be answered for all the session parts.

**Phase 2**

In the last two weeks you have facilitated two sessions. In the first session you focused on thinking about high-risk situations, managing stress, and managing your emotions. In today’s session, you talked about managing problems.

Today I will ask you some questions about your experiences of these two sessions. First of all, I’m interested in the overall experience;

[1.1] Were there parts that you think were helpful for the participants, or not?

Which parts were helpful? And why?

Which parts were not helpful? And why?

[1.2]. Were there parts that you felt are not relevant for people living here/people in similar circumstances as the participants? e.g., because they don’t address the specific problems they experience.

If so, which parts?

Why are these parts not relevant?

[1.3] Did you do anything differently from how it was specified in the manual? If yes, what did you do differently and what was the reason?

|  |
| --- |

[1.4] Was there any strategy or activity that you struggled to explain to the participants?

| If yes, what was it about these strategies or activities that made it difficult to explain?  What changes would you make to make these strategies or activities easier? |
| --- |

[1.5] What strategy or activity did you find easy to explain to participants?

[1.6] Would there be anything that you would change in this session overall? If so, what

changes would you make?

If yes, why would you make these changes?

**Session 3**

In session three you talked to participants about high-risk situations, managing stress, and managing your emotions.

[2.1] What was your experience of delivering and explaining these strategies and activities in

this session? Probe: High-risk situations, managing stress, managing emotions

|  |
| --- |

[2.2] Were you able to deliver the intervention session in the planned time?

|  |
| --- |

**Session 4**

In session four you talked to participants about problem management

[3.1] What was your experience of delivering and explaining that strategy in this session?

|  |
| --- |

[3.2] Were you able to deliver the intervention session in the planned time?

|  |
| --- |

[3.3] What was your experience of using the handout on managing problems?

[3.4] Do you think the handout on managing problems accurately reflects the lessons learned in this session?

4. Are there any additional comments you want to make or issues you want to raise about the PM+A intervention so far?

Thank you.

**TREATMENT COHORT – PHASE II (session 3 and session 4)**

**PM+A Post-Session Debrief: Participant**

[**INSTRUCTIONS**]

These questions are asked by the researcher to the PM+A participant, immediately after each phase. We ask these questions to make sure we understand how people experience the sessions. The questions are about (1) the *relevance* of the session content; (2) *cultural acceptability* of the session content; (3) *comprehensibility* of the session content; and (4) the *perceived effectiveness* of the session content. The questions should be answered for all the session parts.

**Phase 2**

In the last two weeks you have had two sessions. In the first session you focused on thinking about high-risk situations, managing stress, and managing your emotions. In today’s session, you talked about managing problems.

Today I will ask you some questions about your experiences of these two sessions. First of all, I’m interested in the overall experience;

[1.1] Can you describe your overall experience of these two sessions?

[1.2] Have there been any parts in the topics covered in the last two weeks that have particularly stood out to you?

[1.3] Were there parts that you think were helpful for you, or not?

Which parts were helpful? And why?

Which parts were not helpful? And why?

[1.4] Were there parts that you felt are not relevant for people living here/people in similar circumstances as you? If so, which parts? and why?

If so, which parts?

Why are these parts not relevant?

[1.5] Were there any parts that you felt were inappropriate?

In the beginning of session three, you talked about identifying high-risk situations, you practiced managing stress exercises, and managing emotions exercises. I’m going to ask you some questions about each of these.

**Strategy 1**

[2.1] Did you have any trouble understanding how you can identify high-risk situations? (For

example, hanging out with peers, negative emotions such as feeling distressed, sad or

angry, positive emotions such as excitement etc)

If yes, why was it hard to understand?

[2.2] If you had to describe how to identify high-risk situations to a friend, how would you

describe it?

**Strategy 2**

[3.1] Did you have any trouble understanding the managing stress breathing exercise?

[3.2] If yes, why was it hard to understand?

[3.3] What did you think about the handout you were given regarding managing stress/breathing exercise?

[3.4] Do you think the handout reflects the takeaway messages you learned during the session?

If not, why not?

[3.5] If you had to describe the managing stress exercise to a friend, how would you do that?

**Strategy 3:**

[4.1] Did you have any trouble understanding the managing emotions strategy? This is the

exercise where we talked about how stressful events can lead to negative emotions like

anger, or sadness resulting us to drink.

[4.2] If yes, why was it hard to understand?

[4.3] What did you think about the handout you were given regarding managing emotions?

[4.4] Do you think the handout reflects the take away messages you learned during the session?

If not, why not?

[4.5] If you had to describe the managing emotions exercise to a friend, how would you do that?

In today’s session, you talked through a problem management strategy.

**Strategy 4**

[5.1] Did you have any trouble understanding the problem management strategy? This is where we tried to list problems that we are facing in life, dividing them in solvable problems and unsolvable problems, and coming up with solutions for solvable problems.

If yes, why was it hard to understand?

[5.2] What did you think about the handouts you were given regarding problem management?

[5.3] Do you think the handouts reflect the takeaway messages you learned during the sessions?

If not, why not

[5.4] If you had to describe the problem management exercise to a friend, how would you do

that?

6. Are there any additional comments you want to make or issues you want to raise about the PM+A intervention so far?

[**READ OUT LOUD**]

Thank you very much for your answers.

# TREATMENT COHORT – PHASE III (session 5 and session 6)

**PM+A Post-Session Debrief: FACILITATOR**

[**INSTRUCTIONS**]

These questions are asked by the researcher to the PM+A facilitator, immediately after each phase. We ask these questions to make sure we understand how people experience facilitating the sessions. The questions are about (1) the *relevance* of the session content; (2) *cultural acceptability* of the session content; (3) *comprehensibility* of the session content; and (4) the *perceived effectiveness* of the session content. The questions should be answered for all the session parts.

**Phase 3**

In the last two weeks you have had two sessions. In the first session get going and keep doing, and how to strengthen your social support. In today’s session, you talked about staying well and looking forward.

Today I will ask you some questions about your experiences of these two sessions. First of all, I’m interested in the overall experiences;

[1.1] Were there parts that you think were helpful for the participants, or not?

Which parts were helpful? And why?

Which parts were not helpful? And why?

[1.2]. Were there parts that you felt are not relevant for people living here/people in similar circumstances as the participants? e.g., because they don’t address the specific problems they experience.

If so, which parts?

Why are these parts not relevant?

[1.3] Did you do anything differently from how it was specified in the manual? If yes, what did you do differently and what was the reason?

|  |
| --- |

[1.4] Was there any strategy or activity that you struggled to explain to the participants?

| If yes, what was it about these strategies or activities that made it difficult to explain?    What changes would you make to make these strategies or activities easier? |
| --- |

[1.5] What strategy or activity did you find easy to explain to participants?

[1.6] Would there be anything that you would change in this session overall? If so, what changes would you make?

If yes, why would you make these changes?

[1.7] Were you able to deliver the intervention session in the planned time? If not, why not?

Seyi ita kan agder deresu deresa ta musada de fi zaman al ita kan nizaam/ Kuta? Lo

mafi, malu?

**Session Five**

In session five, you talked about get going and keep doing, and how to strengthen your social support.

[2.1] What was your experience of delivering and explaining this strategy in this session?

|  |
| --- |

[2.2] Were you able to deliver the intervention session in the planned time?

|  |
| --- |

[2.3] What was your experience of using the handout on Get Going keep doing?

[2.4] Do you think the handout accurately reflects the lessons learned in this session?

[2.5] Do you think the handout was fully comprehensible to both literate and illiterate participants?

If not, how can we make it more understandable?

**Session 6**

In session six you talked to participants about staying well, how to help others, and looking forward

[3.1] What was your experience of delivering and explaining this strategy in this session?

|  |
| --- |

[3.2] Were you able to deliver the intervention session in the planned time?

|  |
| --- |

[3.3] What was your experience of using the handout?

[3.4] Do you think the handout accurately reflects the lessons learned in this session?

[3.5] Do you think the handout was fully comprehensible to both literate and illiterate participants?

If not, how can we make it more understandable?

[3.6] Overall, after completing the intervention, do you think there is anything else that should be included during or after the intervention?

[3.7] In terms of the intervention delivery, do you think the gender of the facilitator makes a

difference?

[**READ OUT LOUD**]

[3.8] To what extent do you think PM+A could be incorporated into routine practice?

Thank you for participating.

**TREATMENT COHORT – PHASE III (session 5 and session 6)**

**PM+A Post-Session Debrief: Participant**

These questions are asked by the researcher to the PM+A participant, immediately after each phase. We ask these questions to make sure we understand how people experience the sessions. The questions are about (1) the *relevance* of the session content; (2) *cultural acceptability* of the session content; (3) *comprehensibility* of the session content; and (4) the *perceived effectiveness* of the session content. The questions should be answered for all the session parts.

Please make sure you have completed the consent form with the participant.

**Phase 3**

In the last two weeks you have had two sessions. In the first session get going and keep doing, and how to strengthen your social support. In today’s session, you talked about staying well and looking forward. Today I will ask you some questions about your experiences of these two sessions.

[1.1] Can you describe your overall experience of these two sessions?

[1.2] Have there been any parts in the topics covered in the last two weeks that have particularly stood out to you?

[1.3] Were there parts that you think were helpful for you, or not?

Which parts were helpful? And why?

Which parts were not helpful? And why?

[1.4] Were there parts that you felt are not relevant for people living here/people in similar

circumstances as you? If so, which parts? and why?

If so, which parts?

Why are these parts not relevant?

[1.5] Were there any parts that you felt were inappropriate?

In the beginning of session five, you talked about get going and keep doing, and how to strengthen your social support.

**Strategy 1**

[2.1] Did you have any trouble understanding the ‘get going and keep doing’ strategy? This is

the strategy where we talked about activities that we used to liked but don’t do anymore

because of our mood, and that uptake of these pleasant activities may improve our overall

mood and outlook in life.

If yes, why was it was hard to understand?

[2.2] What did you think about the handouts you were given regarding ‘get going and keep

doing’?

[2.3] Do you think the handouts reflect the takeaway messages you learned during the sessions?

If not, why not?

[2.4] Is there anything we can change to make the handout more helpful to you?

[2.5] If you had to describe the ‘get going and keep doing’ strategy to a friend, how would you

describe it?

**Strategy 2**

[3.1] Did you have any trouble understanding the conversation about how to strengthen your

social support? This is where we talked about family, friends, neighbours, and community

groups that can hep us deal with all the things that are going on in life, and can help us feel

connected with people and the community

If yes, what about it was hard to understand?

[3.2] What did you think about the handouts you were given regarding strengthening your social support?

[3.3] Do you think the handouts reflect the takeaway messages you learned during the sessions?

If not, why not?

[3.4] Is there anything we can change to make the handout more helpful to you?

[3.5] If you had to describe how to strengthen your social support to a friend, how would you

describe it?

In today’s session, you talked about staying well and looking forward.

**Strategy 3**

[4.1] Did anything stand out to you in terms of the conversations you had with your facilitator about staying well and looking forward? This is where we talked about how we can stay well in the future and our goals after the programme has ended.

[4.2] Was there anything that you found difficult to understand?

[4.3] Now thinking about everything you have learned in the last 5 to 6 weeks, do you foresee

any barriers using the strategies and lessons you have learned during PM+A in your day to day life?

[4.4] Was there anything you think we should have addressed in the intervention that we didn’t

address?

[4.5] In terms of the intervention delivery, do you think the gender of the facilitator makes a

difference?

Thank you for participating.
